# Editorial: Editor’s Pick 2021: Highlights in Cell Adhesion and Migration

**DOI:** 10.3389/fcell.2022.852781

**Published:** 2022-03-07

**Authors:** Claudia Tanja Mierke

**Affiliations:** Faculty of Physics and Earth Science, Peter Debye Institute of Soft Matter Physics, Biological Physics Division, University of Leipzig, Leipzig, Germany

**Keywords:** cell and matrix mechanics, focal adhesion proteins, actin and actin binding proteins, mechanosignaling, migrasome, microtubules, integrins, individual and collective migration

The Editor’s Picks are not a standard Frontiers in Cell and Developmental Biology Research topic in Cell Adhesion and Migration, as it focuses on outstanding high-impact manuscripts that are of interest to a broad group of scientists and were not already assigned to a specific Research topic. The migration and invasion of cells can be sculpted in various model systems, encompassing human (Cuenca et al., 2020; Hayn et al.; Paradžik et al., 2020; Zhang et al., 2020; Liu et al., 2021; Zhou et al., 2021) and murine cell cultures (Becsky et al.; Cheong et al.; Jiang et al.) or animals, such as zebrafish (Van der Meulen et al.; Dries et al.) and mouse models (Burr et al.; Garcia-Guillin et al.). In this regard, it is crucial to choose an appropriate microenvironment for the analysis of cell adhesion and migration, as there is an interaction between the phenotype of the cytoskeleton, the nuclear or organelle phenotype of the cells and their extracellular matrix environment (Mierke, 2020). This particular research theme of Frontiers in Cell and Developmental Biology Section Cell Adhesion and Migration includes eleven original research articles, one methods article and two reviews.

Burr et al. investigated how the AGE/RAGE signaling pathway alters the migration of two kinds of cardiac fibroblasts, such as non-diabetic and diabetic cardiac fibroblasts (Burr et al.). Notably, the AGE/RAGE signaling pathway has been altered by the supplementation of ERK1/2 and PKC-ζ inhibitors, and by exogenous AGEs treatment. Since the small GTPase Rap1 has been identified to fulfill fundamental cellular tasks, such as cell-cell and cell-matrix adhesion, proliferation and polarity governance (Chrzanowska-Wodnicka et al., 2015), it seems to be likely that it acts in the Age/RAGE signaling cascade. In fact, Burr et al. found that the small GTPase Rap1a functions as a coupler between the extracellular cues to intracellular events.

In 2018, novel adhesive structures referred to as reticular adhesions (RAs) have been detected. They are abundant in integrin αvβ5, while lacking most of the characteristic cytoskeletal moieties seen in other integrin adhesion complexes (IACs), and are interconnected in their place with the clathrin system (Lock et al., 2018). In addition, reduced motility and heightened susceptibility to microtubule toxins (MT), paclitaxel, and vincristine, following transfection with integrin-αV-specific siRNA, has been identified in the melanoma cell line MDA-MB-435S, implying an association between adhesion and medication susceptibility (Stojanović et al., 2018). Paradžik et al. addressed the clarification of the enabling mechanism and identified αV-dependent modifications in the assortment of IACs that compose the adhesome (Paradžik et al., 2020). Mass spectrometry proteomics yielded, that cells preferably exploit integrin αVβ5 for the assembly of IACs. Due to knockdown of the αV-integrin subunit, reduced levels of specific elements of the cortical microtubule stabilization complex that align MTs to adhesion sites can be detected, including the proteins KN motif and ankyrin repeat domain-containing 1 (KANK1) and KANK2. Moreover, the knockdown of KANK2 in human MDA-MB-435S melanoma cells can replicate the phenotype of the integrin-αV knockdown and induce enhanced susceptibility to MT toxicants and diminished migration (Paradžik et al., 2020). Finally, they pointed out that KANK2 is a critical factor that joins integrin-αVβ5 adhesion complexes to MTs and facilitates actin-MT interactions that are vital for both susceptibility to MT toxins and migration of cells.

Besides the adhesome, a new migration-dependent organelle referred to as the migrasome has been identified to facilitate the liberation of cytoplasmic constituents; this cellular process has been termed migracytosis (Ma et al., 2015). Thereafter, the migrasomes can be taken up by adjacent cells. Thus, Zhang et al. reviewed timely the impact of the migrasome in a cardiovascular system and pointed out that the generation of the migrasome is tightly governed through tetraspanins (TSPANs), cholesterol, and integrins. Their review focuses on TSPANs, migrasomes, and migracytosis, which perform critical functions in the coordination of vascular homeostasis. Moreover, the differences between migrasomes and exosomes are pointed out (Zhang et al., 2020).

In a zebrafish model, Van der Meulen et al. intended to analyze the performance of the periocular mesenchyme (POM) and identify transcriptional activities during the early development of the zebrafish anterior segment (AS) of the eye (Van der Meulen et al.). Disruption of AS might be the reason for congenital blindness and disposition to glaucoma. Analysis of the performance of POM cells at fixed time points and in real time has been arranged in a capacious model for the settlement of the AS of the zebrafish. In doing so, they generated transcriptomic single cell microprofiles (scRNA) from the POM-generated reporter lines and mapped unique expression patterns of distinct subpopulations. Subsequently, they noted that AS-associated POM or anterior segment mesenchyme is not uniform, but consists of multiple subpopulations with diverse colonization profiles, migratory habits, and transcriptomic patterns (Van der Meulen et al.).

Efficient cell locomotion demands the polarization of cells, which is well-known to entail the generation of leading and trailing ends, adequate placement of the nucleus, and realignment of the Golgi apparatus and centrosomes with respect to the leading edge (Vicente-Manzanares et al., 2005; Keller-Pinter et al., 2010). Asymmetric calcium gradient (Ca^2+^) evolution from anterior to posterior is also prerequisite for focal adhesion assemblage and actomyosin-driven contractility to govern migration. Becsky et al. identified that knockdown of syndecan-4 results in nanoscale changes in the architecture of lamellipodial actin fibers of motile myoblasts (Becsky et al.). They subsequently revealed that syndecan-4 spreads asymmetrically throughout cell migration and dictates cellular polarity through affecting centrosome location and anterior-posterior Ca^2+^ gradient evolution. Despite several earlier published articles detailing a function of syndecan-4 in cell migration by triggering EMT (Toba-Ichihashi et al., 2016) and subsequently fostering the persistence of locomotion (Becsky et al.), in their original article they provide a super-resolved structural view of the actin cytoskeleton of syndecam-4 knock-out cells. In addition, this is the initial record delineating the involvement of syndecan-4 in the deployment of the Ca^2+^ gradient and the placement of the centrosome in a wandering cell.

The polarization of cells is also important in cellular assemblies, as it affects the morphology of tissues and supports repair mechanisms. Specifically, there is a phenomenon known as planar cell polarity (PCP), which is part of the non-canonical Wnt signaling pathway and governs the group polarization of cells at the level of a cell layer. Even though PCP modulates actin cytoskeleton rearrangements through its downstream effector RhoA (Henderson et al., 2018), suggesting a function of the PCP pathway in mechanotransduction processes where mechanical signaling sensing quickly elicits signaling mechanisms that result in cytoskeleton rearrangement and alteration of cell shape or movement, which had not yet been established. Mechanical stress triggers activation of RhoA, which encourages actin stress fiber generation and promotes phosphorylation of myosin light chain 2 through Rho-associated kinase (Sun et al., 2016). Cheong et al. hypothesized that VANGL2 may serve a key purpose in mechanosignaling, which they set out to test in a mouse model (Cheong et al.). The hypothesis is based on a prior investigation that demonstrated a connection between the PCP/WNT5A-Frizzled DVL pathway and YAP (Park et al., 2015). Yap is a well-known mechanotransducer that translates mechanical inputs into biochemical responses through the regulation of gene expression (Jaalouk and Lammerding, 2009). Because YAP and the looptail mouse mutant (Vangl2Lp) deficiency result in similar defects in lung branching (Lin et al., 2017), it seems probable that Vangl2 may act in cellular mechanotransduction. In particular, the Vangl2Lp has the missense mutation S464N in the Vangl2 gene. Vangl2Lp represents a dominant-negative type of mutation affecting not just the trafficking of mutant VANGL2 from the endoplasmic reticulum to the Golgi apparatus, but also blocking the trafficking of wild-type VANGL2 protein to the plasma membrane, causing a complete loss of function (Kibar et al., 2001). Using Vangl2Lp, Cheong et al. therefore investigated whether VANGL2 malfunction alters cellular mechanics and mechanotransduction processes. They established that VANGL2 is necessary for normal alveologenesis and wound healing mediated through its involvement in directional cell migration. They mechanistically demonstrated that disruption of VANGL2 impairs focal adhesion complexes, stress fiber generation, and activation of MLC2, which results in impaired intracellular contractility through the RhoA signal pathway. These abnormalities lead to disturbed generation of traction forces and a lack of the mechanotransducer YAP (Cheong et al.).

In a zebrafish model, Dries et al. explored the collective migration of cells (Dries et al.). Specifically, the zebrafish is a singular model for the analysis of collective cell migration, axonal growth, and route-finding mechanisms. It is recognized that the posterior lateral line system (pLLS) of these aquatic species consists of small clustered mechanosensory organs located longitudinally down the side of the animal, which evolve from proneuromasts (Metcalfe et al., 1985). Dries et al. revealed that the neuronal cell adhesion molecule Ncam1b forms an integral element of the pathways that induce and govern the evolution of pLLS in zebrafish. Specifically, they inferred that morpholino knockdowns of Ncam1b firstly, decrease cell proliferation inside the primordium, secondly, decrease Fgf target gene Erm expression, thirdly, profoundly impair proneuromast assembly, and fourthly, interfere with primordium migration. Their findings aimed in the following model: Firstly, Ncam1b becomes expressed at the trailing zone of the posterior lateral line primordium. Thereby, it directly interferes with Fgfr1a to trigger the transcription factor Erm expression. Consequently, Erm fosters the proliferation of cells within the trailing zone. However, the proliferation within the leading zone is guided through the Wnt pathway by the transcription factor Lef1. Second, Ncam1b elicits expression of the chemokine receptor Cxcr7b. This could be attributable to direct Fgfr1a signaling on the one hand or to Fgfr1a-dependent inhibition of Wnt signaling on the other hand, which ends up blocking the expression of Cxcr7b. Cxcr7b labels the trailing zone where proneuromasts decelerate to be ultimately segregated. Thirdly, homophilic binding of Ncam1b between adjacent cells effectively steadies the newly established proneuromasts. Ncam1a, on the contrary, is not decisive for the development of pLLS. This argues for a kind of sub-functionalization of the two Ncam1 paralogs in zebrafish, as suspected previously (Langhauser et al., 2012). Finally, Ncam1b acts as a new type of actor in the intricate loopback network that governs pLLS assembly (Dries et al.). Subsequently, it influences several cellular tasks, including primordial cell proliferation, the locomotion of a collective of cells and the abundance of sensory proneuromasts.

Concentrating on the mechanical features of cell migration and invasion and depicting the heterogeneity of extracellular matrices, Mierke’s review discusses that there are three different directions of cell motility centered on the mechanical facets (Mierke, 2020). In the first place, the generally deployed invasion assays are discussed where structural and structure-based environmental mechanical stimuli are exploited; in the second place, the mechano-invasion assay is presented where cells are examined to be influenced by mechanical forces to cause migration and invasion; and in the third place, the impact of cell mechanics, comprising cytoskeletal and nuclear mechanics, is mentioned to interfere with motile and invasive cells. Apart from that, what also arises is the interplay between the cytoskeleton of the cell and its constituent compartments, for example, the cell nucleus. Specifically, a ternary approach is outlined to examine the effects of mechanics on cell migration and invasion by incorporating the impacts of the mechano-phenotype of the cytoskeleton, nucleus, and the microenvironment of the cell throughout the spectrum of analysis (Mierke, 2020). The final target still appears to rely on tunable mechanical excitations delivered to 3D matrices with diverse biochemical and embedded cellular assemblies. Future research will also emphasize co-culture efforts in which surrounding cells, such as cancer-associated fibroblasts or macrophages and cancer-associated endothelial cells, can promote or interfere with cancer cell migration and invasion by perhaps modifying the mechano-phenotype of cancer cells, comprising the cytoskeleton as well as the mechano-phenotype of the nucleus and other organelles, or the extracellular matrix’s mechano-phenotype.

There is a major commitment of cell motility research toward three-dimensional (3D) cell migration. However, 3D motility has been preferably examined using biomimetic extracellular matrix models. Hayn et al. pointed out that in most of these investigations, *in vitro* collagen scaffolds are presumed to be homogeneous because they are usually composed of a particular type of collagen, e.g., type I collagen that has been isolated from a single species (Hayn et al.). The impact of any local changes in extracellular matrix microstructure on matrix mechanics and migration of cells is poorly understood. Hayn et al. postulate that there are local inhomogeneities that alter cell migration because of changes in matrix mechanics, which are common in *in vivo* tissue scaffolds and have even been implicated in abnormal tissues, such as cancer. To dissect the implications of structural inhomogeneities on cell locomotion, Hayn et al. utilized a blend of rat tail and bovine skin type I collagen, along with pure rat and bovine collagens at various concentrations to evaluate 3D inhomogeneities of the architecture. Actually, the invasiveness of three breast cancer cell types is modified by the type and concentration of the matrix, indicating that these two determinants are decisive for the invasiveness of the cells (Hayn et al.). Their outcomes demonstrated that the local inhomogeneity of the matrix framework is one more critical factor to account for the disparities in cell migration, which is not solely a function of the pore size and the stiffness of the collagen matrices. Consequently, these three different biophysical constraints permit us to accurately define the structure and mechanics of the collagen matrix-based migration model schemes.

The interpeduncular nucleus is a hindbrain structural component consisting of three main areas: the prodromal area (Pro), situated at the isthmus (Ist), and the rostral and caudal interpeduncular domains (IPR, IPC) in rhombomere 1 (r1). Garcia-Guillin et al. investigated the impact of the netrin-1/DCC signaling pathway on these migrations, as it is widely acknowledged to govern distinct processes of neuronal migration across the brain (Garcia-Guillin et al.). Specifically, Garcia-Guillin et al. studied the evolution of the interpeduncular nucleus in wild-type and Deleted in Colorectal Cancer (DCC)−/− mice during late gestational ages. *In situ* hybridizations were primarily performed to pinpoint cells expressing each of the listed genes above, these genes comprise the following transcription factors: Nkx6.1, Otp, Otx2, Pax7, and/or Irx2. Garcia-Guillin et al. discovered that the migration of Nkx6.1^+^ and Irx2^+^ cells into the Pro domain suffered severe impairment by the loss of DCC, as during the process of locomotion of Pax7^+^, Irx2^+^, and Otp^+^ cells that usually dominate the IPR. They also found a slight interference with the migration of the Pax7^+^ and Otx2^+^ cells that build the IPC. Subsequently, they concluded that their findings are not only relevant for developing knowledge of this structure, but also provide a foundation for exploring the potential neuroembryological etiology of brain dysfunctions associated with the habenulo-interpeduncular system.

In their methods article, Cuenca et al. presented a single-cell movement in a modular scaffold that can be employed to model the collective response of glioblastoma cells, the most common and virulent primary brain cancer (Cuenca et al., 2020). Cuenca et al. utilized the human primary glioblastoma cell line U87, that can be cultured as monolayers or tightly spatially grouped and arranged in 3D structures, for example spheroids. Their inclusive model takes into account the main mechanisms implicated in cell migration: chemotaxis of attractants, mechanical perceptual exchanges, and incidental movement. Moreover, their simulations matched and replicated the evolving behavior of the spheroids in a series of migration tests that monitored the trajectories of single cells. From both physiological, e.g., the generation of organs, tissue regeneration, and others, and pathological viewpoints, it is necessary to provide a multifaceted tool that can predict the individual and collective behavior of U87 cells (Cuenca et al., 2020), but it can also anticipated to be adaptable to a variety of settings, e.g., different cell types and culture conditions.

Zhou et al. endeavor to investigate diversely expressed long noncoding RNAs (lncRNAs) and mRNAs involved in atherosclerosis (Zhou et al., 2021). They then evidenced the expression of lncRNAs employing quantitative real-time polymerase chain reaction and coexpressed targeting genes in established proliferation and migration model systems of human coronary artery smooth muscle cells (HCASMCs). Specifically, oxidized low-density lipoprotein (ox-LDL) has been added to the culture medium of HCASMCs in order to trigger their proliferation and migration. 68 lncRNAs and 222 mRNAs have been identified that are differentially expressed in atherosclerosis and act as candidates that play critical roles in atherosclerosis (Zhou et al., 2021). Database analysis revealed that the Fanconi anemia signaling pathway could potentially be implicated in atherosclerosis. A sum of six coexpressed mRNAs have been found to be grossly upregulated in HCASMCs. Zhou et al. hypothesized that the variously expressed lncRNAs detected through RNA sequencing and confirmed in smooth muscle cells could serve as a regulatory therapeutic target for HCASMC proliferation and migration in atherosclerosis, thereby representing a new diagnostic ground and a therapeutic objective for the cardiovascular disease setting. Finally, their work appears to offer a new perspective on ox-LDL-induced HCASMC dysfunction in atherosclerosis and potentially proposes a promising coronary artery disease therapeutic strategy, encompassing a new diagnostic rationale and therapeutic objective.

Activin A belongs to the transforming growth factor-beta (TGF-β) superfamily and operates in the process of tissue healing and fibrosis. The cytokine signaling transduction cascades are governed by activin A supporting the inflammatory reaction (Jones et al., 2007). Moreover, activin A controls the activation of M2 macrophage to interfere with the wound healing process after tissue injury and fibrosis during the late inflammatory stage (Morianos et al., 2019). Jiang et al. examined the effect of activin A on adhesion and migration employing the human L929 fibroblast cell line. They found that activin A promotes fibroblast migration using transwell chambers and microfluidics (Jiang et al.). In addition, activin A causes the expression of α-SMA and liberates TGF-β1, factors intimately associated with tissue fibrosis. Activin A increased calcium content in L929 cells and p-ERK protein activity. As a consequence, their outcome data implied that activin A, as a new kind of chemokine, triggers chemotactic migration of L929 cells using ERK signaling and subsequently contributes to fibrosis (Jiang et al.).

The dynamic actin cytoskeleton governs the process of cell locomotion in a spatial and temporal manner (Tang and Gerlach, 2017). Cell migration is preceded by the establishment of actin-based plasma membrane protuberances at the front line of migrating cells (Lauffenburger and Horwitz, 1996). There, a dynamic conversion of the branched actin filament reticulum into lamellipodia takes place, which is accompanied by multiple actin regulators, such as actin-related protein 2/3 (Arp2/3), and filamins, including filamin A (Skau and Waterman, 2015). Liu et al. examined the regulation of filamin A stability (Liu et al., 2021). In the filamentous fungus Aspergillus nidulans, the developmentally conserved nuclear distribution gene C (NudC) has been revealed to be an upstream element of NudF, a key dynein regulator that affects nuclear locomotion (Jheng et al., 2018). It is possible to propose that NudC could improve the folding of its candidate proteins through its proprietary chaperone activity or its function as an Hsp90 cochaperone through volatile modification of Hsp90 ATPase activity (Dean and Johnson, 2021). Liu et al. further identified that NudC-L279P, a conserved mutation of human NudC to Leu146, resulting in reduced NudF in Aspergillus, compromises NudC function and affects Hsp90 chaperone activity (Zhu et al., 2010). Overexpression of NudC-L279P reduces LIS1, a major effector of cell migration, on a Hsp90-driven basis (Zhu et al., 2010). Nevertheless, the undergirding mechanism of NudC in modulating mammalian cell migration remarkably stays obscure. Liu et al. have demonstrated that NudC can stabilize filamin A due to their interplay. NudC L279P overexpression disturbs filamin A and inhibits cell locomotion. In summary, ectopic expression of Hsp90 reverts filamin A fragility and phenotype aberrations conferred upon by overexpression of NudC-L279P. Therefore, Liu et al. propose that the NudC-L279P mutation works to destabilize filamin A via inhibiting the Hsp90-facilitated chaperoning route, which is a yet to be delineated mechanism that is pivotal in governing filamin A stability.

All articles show the complexity of different cell adhesion and migration research approaches, whereby vastly different model systems have been employed to reveal the biochemical and mechanical characteristics of cells, spheroids, tissues and entire organisms. There are still many more interesting facts to explore, such as whether there is a unique mechanical phenotype of cells, tissues and their microenvironment independent of the model organism that fosters the motility of cells or collections of cells. The broad variety of techniques and methods employed in the different investigations indicates that this is highly needed to generate a comprehensive view on cell adhesion and migration.
